# Intra-pulse beam steering in a mid-infrared quantum cascade laser

**DOI:** 10.1007/s11082-014-0006-0

**Published:** 2014-08-24

**Authors:** Emilia Pruszyńska-Karbownik, Kazimierz Regiński, Piotr Karbownik, Bohdan Mroziewicz

**Affiliations:** Institute of Electron Technology, al. Lotników 32/46, 03-668 Warsaw, Poland

**Keywords:** Beam steering, Quantum cascade laser, Far field

## Abstract

The intra-pulse measurements of the beam steering in an AlGaAs/GaAs quantum cascade laser are presented in this paper. The experimental results are explained by a two-mode theoretical model. The near field of the laser radiation is calculated according to the effective index method and transposed to the far field numerically according to Huygens principle. The maximal observed value of deflection of the beam has been found to be $17^{\circ }$. For supply currents in the range from 1.6 to 2.4 times the threshold the beam steering occurs only on one side of the resonator axis, and stays is the same for all current values. For higher supply current, it occurs alternately on both sides and exhibits a bistability. The time period of the beam direction change has been found to be about 40 ns for the lowest current and was decreasing with the current increase to about 20 ns.

## Introduction

Quantum cascade lasers emitting in the mid-infrared band are valuable sources for molecular spectroscopy, telecommunication and medicine. Methods of their fabrication are currently relatively mature. They work at high temperatures, under continuous wave regime and can deliver high output power. However, to make them fully useful , it is required to assure that they emit stable and well-shaped output beams. This in practice might be difficult because of the so called beam steering which is a phenomenon manifested by changing direction of the beam emission. This phenomenon occurs in various types of semiconductor lasers including quantum cascade lasers. In the latter the beam steering was already observed upon change of the driving current (Bewley et al. 2005; Yu et al. 2009; Yang et al. 2012) and also when the supply pulse was of a constant value (Yu et al. 2012).

Research on the intra-pulse beam steering was based so far on comparing two parts of the driving current pulses that were several tens of nanoseconds long (Yu et al. 2012). We will explore dynamics of this phenomenon on a scale of single nanoseconds.

The asymmetry of the spatial power distribution in the far field must arise from interference of two or more transverse mode of the same frequency. Should the modes be of different frequencies, they would not interfere and the far field distribution would be symmetrical even if the near field was asymmetrical. That has been confirmed experimentally by Yu et al. (2012) who published spatially dependent spectra of the steered beams and demonstrated frequency locking between transverse modes.

The origin of the beam steering phenomenon has been thus ascribed to a lateral hole burning in the population inversion. Such an idea in respect of carrier distribution was analysed in detail by Yang et al. (2012). Keeping this in mind, in this paper we will focus on an optical analysis of the coexistence of the transverse modes and its variation in time.

## Experiment

The investigated device was an $\hbox {Al}_{0.45}\hbox {Ga}_{0.55}\hbox {As/GaAs}$ quantum cascade laser, the details of which are described in Kosiel et al. (2011). The laser chip was processed to obtain a double trench mesa with width of $25\,\upmu \mathrm {m}$, and then was cleaved into cavities 2 mm long. It was subsequently mounted epilayer-down on a gold galvanized copper submount. The wavelength of the emitted radiation was $\lambda =9.4\,\upmu$m.

**Fig. 1:**
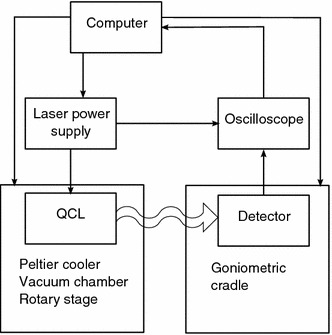
Simplified diagram of the experimental set-up

The diagram of the experimental set-up is shown in Fig. 1. The studied laser is placed on a Peltier cooler in a vacuum chamber which is located on a rotary stage. The submount temperature has been set on 260 K. The laser was supplied with 100 ns pulses and frequency of 500 Hz. The pulse rise and fall times were 15 and 10 ns, respectively. Laser radiation leaves the chamber through a 2 in. ZnSe window. Because the window slightly shrinks the beam, we have made relevant mathematical corrections of the measurement results to compensate this effect (Pruszyńska-Karbownik et al. 2013). The laser radiation was recorded with the TE cooled HgCdTe detector which is located on a goniometric cradle described in Pruszyńska-Karbownik et al. (2013). The signal of the detector has been recorded point by point by a computer using an oscilloscope. The signal loading from the oscilloscope is being triggered together with the beginning of the supply pulse. Then it is read at regular intervals of 1 ns, starting from the time point when the pulse rise ends. It allows to estimate the changes of the optical power and the beam shape occurring statistically during pulses.

The measurement of the far field along the slow axis is performed by rotating the stage with the laser chamber, while along the fast axis by moving the goniometric cradle with the detector.

## Theory

**Fig. 2:**
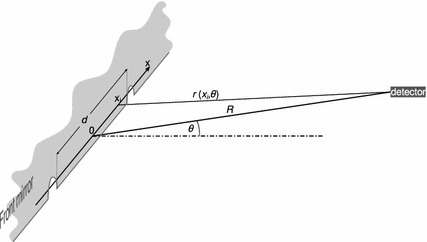
Schematic diagram of the model geometry

A schematic diagram of the theoretical model geometry is presented in Fig. 2.

For calculation of the near field we use the effective index method and consider only the slow axis direction. In this direction the active region is confined by trenches filled in by metal. Therefore, the field distribution $E_k(x)$ of the kth mode can be expressed by the formula (Szymański et al. 2013):

1 $$\begin{aligned} E_k(x)=E_{k0}\sin {\frac{(k+1)\pi x}{d}};\quad k=0,1,\ldots , \end{aligned}$$

where $x$ is the coordinate along the slow axis, $d$ is the active region width and $k$ is the number of the mode. The formula is an analytical solution of the Helmholtz equation for such simplified structure.

In our model we consider two first modes with maximal values of $E_{00}$ and $E_{10}$ and with phase difference $\phi$. We assume that the modes are frequency locked. Then the near field distribution is

2 $$\begin{aligned} E(x)=E_{00}\sin {\frac{\pi x}{d}}+E_{10}\sin {\frac{2\pi x}{d}}\cdot e^{i\phi }. \end{aligned}$$

The far field for angle $\theta$ and distance $R$ is calculated from the near field directly by the formula of Huygens principle (Hodgson and Weber 2005):

3 $$\begin{aligned} E(\theta )=C \int _{-\infty }^{\infty } \frac{E(x)}{r(x,\theta )} e^{-i \frac{2 \pi }{\lambda } r(x,\theta )}\,dx, \end{aligned}$$

where $C$ is a proportionality factor and $r(x,\theta )$ is the distance between the source point with the coordinate $x$ and the point at which we determine the far field.

4 $$\begin{aligned} r(x,\theta )=\sqrt{R^2-2Rx\sin {\theta }+{x}^2}, \end{aligned}$$

where $\theta$ is the angle between the axis perpendicular to the laser front mirror and the straight line that connects the centre of the active region and the far field point.

Intensities of the near and far field are linked to the field amplitudes by the formula

5 $$\begin{aligned} I=\frac{1}{2} c n \epsilon _0 \left| E\right| ^2, \end{aligned}$$

where $n$ is the effective refractive index, $c$ is the speed of light in vacuum and $\epsilon _0$ is the vacuum permittivity.

**Fig. 3:**
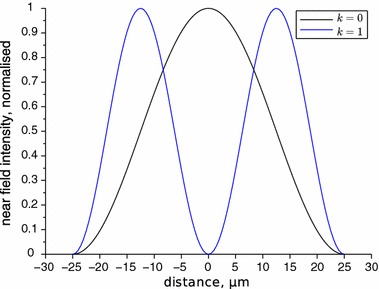
Calculated normalised near field intensities for the first two modes

**Fig. 4:**
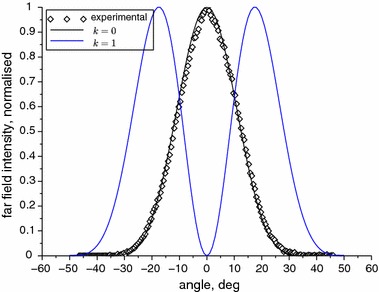
Calculated normalised far field intensities for the first two modes and normalised far field intensity measured near threshold

All the near and far field calculations are made numerically using a program written in Scilab. Figures 3 and 4 present the calculated near and far fields for pure 0th and 1st mode, respectively. In Fig. 4 the experimental near-threshold far field intensity is added for comparison. In this condition only the fundamental mode is induced. We can see that the results of the calculations match to the experiment almost perfectly. We consider this as a proof of the numerical correctness of the used algorithm and as an indication that the model we applied is also correct because not only the shape of the beam but also its divergence angle comply together. To adjust the calculations to the experimental data for a multimode regime, the least squares method for three variables, $E_{00},\,E_{10}$ and $\phi$, has been used.

## Results and discussion

The threshold current for the studied laser in the temperature of 260 K is 6 A. The characteristic temperature $T_0$ of the laser in temperature range of 260–310 K is $111 \pm 7\,\mathrm {K}$. The maximal work temperature of the studied laser is 313 K. The beam shape along the fast axis does not change during the pulse and does not depend on the current. Therefore we focus only on the results for slow axis.

**Fig. 5:**
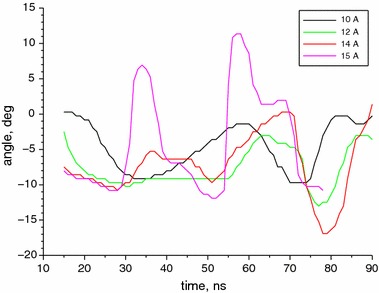
Time dependence of the beam direction angle along the slow axis for different values of current

We have measured the far field of the laser for currents from 7 to 15 A with the step of 1 A. No beam steering has been observed for the current lower than 10 A. Also no such effect has been observed in the temperature higher than 260 K. At 260 K and for currents in the range from 10 to 14 A the beam is steered on one side, while for the current of 15 A, the beam steering alternately on both sides has been observed. The change of the beam direction for several currents in the range from 10 to 15 A is presented in Fig. 5. The graphs for 11 and 13 A are omitted to preserve the clarity of the figure and because their shapes are similar to the ones adjacent.

**Fig. 6:**
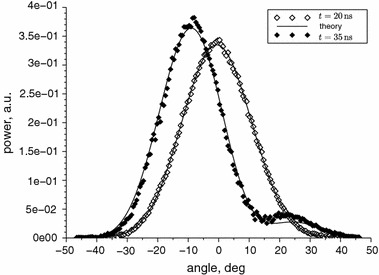
Comparison of beam shape without steering, measured 20 ns after the start of the pulse, and with beam steering, measured 35 ns after the start of the pulse, for the current of 10 A. Theoretical solutions found by using the least squares method are drawn by *solid lines*

The maximum range of the beam steering changes from $10^{\circ }$ for 10 A to $17^{\circ }$ for 14 A. The time period of the beam direction oscillations is about 40 ns for the lowest current and decreases with the current to achieve about 20 ns for 15 A. For all the time points, the theoretical two-mode solutions have been found. Exemplary graphs of these solutions for the stable and steered beam are presented in Fig. 6. As shown, the theoretical solution that was found is close to the experimental data, but it does not exactly reflect the shape of the second lobe of the beam. This could be improved by assuming existence and adding an another higher-order mode to the model, but in our opinion this is not necessary, and the two-mode model is sufficient for our needs (Fig. 6).

For the highest current of 15 A, the dominant mode is initially also the 0th order. Then, the amplitude ratio of the modes varies, and after 80 ns the 0th mode temporarily almost disappears. Unlike for the lower currents, the phase difference changes abruptly,within the time lower than 1 ns, and the most frequently takes the value of $\pm 1.57\,\pi$. The presence of such change in the beam direction was noticed by Bewley et al. (2005) and explained as an accidentally induced by small mode perturbations although did not excluded the mode interference. Our measurements show that this is a bistable system, such as it was suspected in Bewley et al. (2005).

**Fig. 7:**
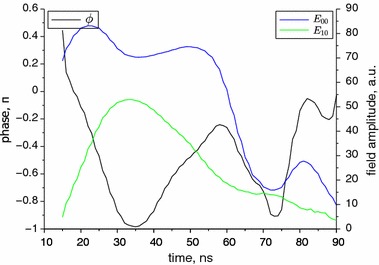
Numerically found phase difference angle and field amplitudes of 0th and 1st modes for current of 10 A

**Fig. 8:**
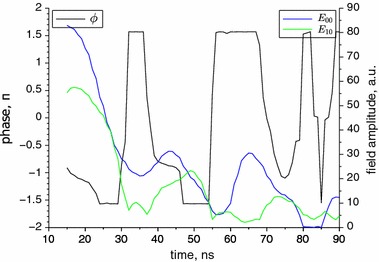
Numerically found phase difference angle and field amplitudes of 0th and 1st modes for current of 15 A

Figures 7 and 8 present the numerically found theoretical solutions of the mode structure during the pulse for the lowest and highest supply current, respectively. For the lowest current with the beam steering present, which is 10 A, during the whole time, the 0th mode is dominant during the whole time. The extreme value of the phase difference is about $\pi$. All the parameters vary in a continuous manner.

## Summary

We have observed the beam steering along the slow axis of the quantum cascade laser beam for the currents $>$1.6 times the threshold. We have shown that the beam steering in quantum cascade lasers is a dynamic phenomenon with the change time of a nanosecond scale but also occurs repeatedly in a similar way in subsequent pulses and the use of the statistical method is therefore justified. With the increase of the supply current, increases not only the beam direction angle but also the rate at which the angle variation takes place. For the supply current that is equal to 2.5 times the threshold, the beam steering occurs alternately on both sides of the resonator axis, exhibiting the bistability, while for the lower currents it occurs only on one side, and stays the same for all current values.

We used a two-mode model to describe the beam steering phenomenon. Addition of an another mode to the model would slightly improve the results of calculations but would also increase dramatically the calculation time. In our opinion, the two-mode model is sufficient to describe the beam steering in the studied laser, but in other structures it may require addition of one or more higher-order modes. However, we have shown that even such a very simple model gives results well matched to the experiment.

Undoubtedly the theoretical model could be greatly improved by taking into an account not only the interfering modes, but also additional modes with different frequencies. Neglecting the noninterfering modes can make the calculated phase differences and the second mode contribution underestimated. To take these modes into account, additional measurements of the spatially dependent spectra for the studied laser have to be performed, which is what we are planning for the near future.
